# Therapeutic potential of systemic brain rejuvenation strategies for neurodegenerative disease

**DOI:** 10.12688/f1000research.11437.1

**Published:** 2017-08-01

**Authors:** Alana M. Horowitz, Saul A. Villeda

**Affiliations:** 1 Biomedical Sciences Graduate Program, University of California San Francisco, San Francisco, California, 94143, USA; 2 Department of Anatomy, University of California San Francisco, San Francisco, California, 94143, USA; 3 The Eli and Edythe Broad Center of Regeneration Medicine and Stem Cell Research, University of California San Francisco, San Francisco, California, 94143, USA

**Keywords:** neurodegenerative disease, brain rejuvenation, caloric restriction, exercise, blood plasma administration, healthspan

## Abstract

Neurodegenerative diseases are a devastating group of conditions that cause progressive loss of neuronal integrity, affecting cognitive and motor functioning in an ever-increasing number of older individuals. Attempts to slow neurodegenerative disease advancement have met with little success in the clinic; however, a new therapeutic approach may stem from classic interventions, such as caloric restriction, exercise, and parabiosis. For decades, researchers have reported that these systemic-level manipulations can promote major functional changes that extend organismal lifespan and healthspan. Only recently, however, have the functional effects of these interventions on the brain begun to be appreciated at a molecular and cellular level. The potential to counteract the effects of aging in the brain, in effect rejuvenating the aged brain, could offer broad therapeutic potential to combat dementia-related neurodegenerative disease in the elderly. In particular, results from heterochronic parabiosis and young plasma administration studies indicate that pro-aging and rejuvenating factors exist in the circulation that can independently promote or reverse age-related phenotypes. The recent demonstration that human umbilical cord blood similarly functions to rejuvenate the aged brain further advances this work to clinical translation. In this review, we focus on these blood-based rejuvenation strategies and their capacity to delay age-related molecular and functional decline in the aging brain. We discuss new findings that extend the beneficial effects of young blood to neurodegenerative disease models. Lastly, we explore the translational potential of blood-based interventions, highlighting current clinical trials aimed at addressing therapeutic applications for the treatment of dementia-related neurodegenerative disease in humans.

## Introduction

**Figure 1.**
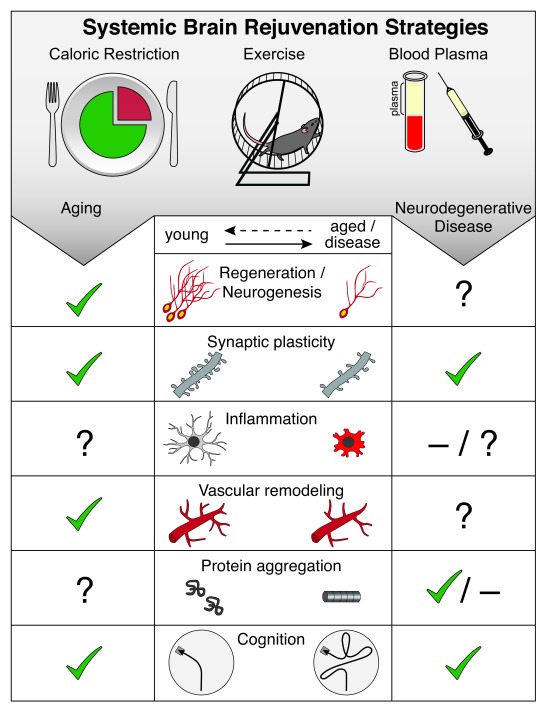
Systemic brain rejuvenation strategies. Hallmarks of brain aging amenable to rejuvenation (middle panel) include decreased regenerative capacity (neurogenesis), impaired synaptic plasticity, increased inflammation, vascular remodeling, increased protein aggregation, and impaired cognitive function. Systemic interventions (top panel), such as caloric restriction, exercise, and blood plasma administration, have been shown to rejuvenate hallmarks of brain aging (left panel) and ameliorate exacerbated pathology in models of neurodegenerative disease (right panel). Cellular or functional rejuvenation elicited by systemic interventions is denoted by a check (✔), lack of rejuvenation is denoted by a dash ( **–**), and yet-to-be-determined effects are denoted by a question mark (?).

Aging is the major risk factor for dementia-related neurodegenerative disease in the elderly. In the brain, aging is accompanied by a series of cellular and functional impairments that collectively drive vulnerability to neurodegenerative disease ( Figure 1). It is important to take into account that, along with cellular and functional decline in the aged brain, organismal aging is broadly associated with global “hallmarks of aging” across all tissues in the body, such as stem cell dysfunction, genomic and protein instability, and altered intracellular communication, to name a few ^1^. Consequently, age-related vulnerability to neurodegenerative disease occurs on a background of organismal-level functional decline, and as such interventions to counteract this vulnerability in the aged brain will benefit from a more systemic approach.

In the United States, approximately 15% of the population, 46 million people, are over the age of 65. The number of older adults is predicted to increase to 24% of the population, about 98 million people, by 2060 ^2^. Unfortunately, this increase in lifespan has been accompanied by a drastic rise in age-associated disease. For example, Alzheimer's disease, one of multiple dementia-related neurodegenerative diseases primarily seen in adults over the age of 65, affected 5 million people in 2013 and is predicted to affect 14 million by 2050 ^3^ in the United States. Beyond the personal cost of living with dementia-related neurodegenerative diseases, the monetary cost to society is in the billions. Thus, while longer lifespan indicates successful scientific and medical progress, it requires a corresponding increase in healthspan, the years lived free from disease, to prove truly transformative for human quality of life. The capacity to rejuvenate the aged brain is emerging as a tantalizing prospect for the treatment of dementia-related neurodegenerative diseases of aging ^4^. Results from animal studies involving systemic interventions that promote healthspan—including a decrease in caloric intake without malnutrition also referred to as caloric restriction (CR), exercise, and exposure to a young systemic environment—demonstrate the rejuvenation of regenerative and functional capacity in aged tissues ( Figure 1). It is now evident that such rejuvenation even extends to the aged brain at the regenerative, functional, and cognitive level. Therefore, the application of proven systemic interventions that extend healthspan may prove key in our ability to restore cellular and functional decline in the aging brain to prevent onset, or even counteract the progression, of dementia-related neurodegenerative disease in the elderly.

In this review, we will focus on blood-based brain rejuvenation strategies and their application to animal models of normal aging and neurodegenerative disease. We will discuss future therapeutic applications to brain rejuvenation and highlight ongoing clinical trials applying these findings to human disease.

## Cellular and functional hallmarks of brain aging

Major hallmarks of brain aging include decreased regenerative capacity, altered vasculature, increased neuroinflammation, and impairments in synaptic plasticity that culminate in cognitive dysfunction ( Figure 1) ^5– 7^. Neurodegenerative disease exacerbates these aging-associated cellular and functional hallmarks and introduces gross cellular loss, archetypal of neurodegeneration ^8^. Here we provide a brief overview of key hallmarks of brain aging.

Regenerative capacity in the brain is mediated by the generation of new neurons from neural stem cells, a process known as neurogenesis, which precipitously declines with age ^9– 11^. Neurogenesis predominantly occurs in two neurogenic zones: the dentate gyrus of the hippocampus ^12^, a region involved in spatial and episodic learning and memory, and the subventricular zone lining the lateral ventricles ^13^. Owing to known associations between adult neurogenesis and cognitive function, age-related loss in regenerative capacity has been proposed to contribute to cognitive decline, although the direct link between the two processes in the context of aging has yet to be resolved ^14, 15^. Neurogenesis is tightly linked to nutritive sources provided by the cerebrovasculature through the blood–brain barrier, an anatomical separation between the central nervous system and the periphery consisting of the vascular cells and glia. With age, cerebral blood flow and blood–brain barrier integrity also decline, compromising metabolic support for neurogenesis and proper neuronal signaling and promoting inflammatory responses by resident immune cells ^16, 17^. Correspondingly, increased activation of astrocytes and microglia underlies the neuroinflammatory hallmark of brain aging, accompanied by increased pro-inflammatory cytokine production ^8^. Such pro-inflammatory changes have now been shown to contribute to vulnerability and advancement of neurodegenerative disease through processes such as accumulation of complement factors (a component of innate immunity) in microglia ^8, 18^. At a functional level, synaptic plasticity also declines with age ^19^. For example, electrophysiology studies reveal an age-related decline in long-term potentiation (LTP), an electrophysiological measure correlated with learning and memory. Functional impairments are also accompanied by corresponding molecular changes in the expression of plasticity-related factors such as immediate early genes. In the context of neurodegenerative disease, an exacerbated decline in synaptic plasticity occurs ^20^. Consistent with synaptic dysfunction, cognitive processes such as learning and memory also decline with age. Neurodegenerative disease magnifies all hallmarks of brain aging while also promoting the accumulation of damaged or misfolded protein aggregates and gross neurodegeneration ^8^, which collectively exacerbate cognitive impairment ^21^.

## Systemic interventions: caloric restriction, exercise, parabiosis, and healthspan

Multiple lines of evidence in animal models point to the malleability of lifespan ^22– 31^. The initiation of rejuvenating interventions functioning at the systemic level, for example CR, in later stages of life was first demonstrated to improve the overall lifespan of an aged organism over 30 years ago ^32– 34^. More recently, systemically mediated rejuvenating interventions such as CR, exercise, and heterochronic parabiosis (in which the circulatory systems of a young and old animal are joined) have succeeded in rejuvenating aged tissues—including muscle, liver, heart, pancreas, bone, spinal cord, and brain—to improve organismal healthspan ^35– 42^.

### Caloric restriction

The most robust and reliably replicated systemic intervention to improve lifespan and/or healthspan in rodents and non-human primates is CR ^43, 44^. In primates, CR has proved to be protective against the development of neoplasia, cardiovascular disease, and glucoregulatory impairment as well as gray matter loss in the brain ^45^. In rodent models, CR leads to beneficial metabolic changes, protection from oxidative stress, neurotrophic factor production, increased autophagy, and neurogenesis ^46– 49^. CR also promotes the maintenance of cerebral blood flow and white matter integrity with age ^50, 51^. While CR has been demonstrated to improve spatial learning and memory in aged mice ^51^, global effects on cognition remain unresolved, as CR failed to improve other hippocampal-dependent cognitive processes ^52– 54^. It is suggested that such inconsistent results could be due to the varied macronutrient ratios in CR regimes, the age of CR onset, and the metabolic heterogeneity of the diverse cell types in the hippocampus ^55– 57^. Nevertheless, CR reliably improves overall organismal healthspan and likewise improves behavioral and cognitive metrics in animal models of neurodegenerative disease ^58, 59^. While the potential benefits of CR for human healthspan are evident, limitations of feasibility, adequate health monitoring, and adherence remain. Indeed, in a long-term CR study conducted by the CALERIE Research Group, low adherence decreased the average caloric deficit from the anticipated 25% to 11.7% over a 2-year period, offsetting major expected beneficial metabolic outcomes ^60^. Consequently, limits to the application of CR need to be further addressed before widespread therapeutic application.

### Exercise

Robust benefits of exercise have also been consistently observed to increase healthspan, although its role in extending lifespan in mice remains obfuscated ^61– 63^. Exercise has been shown to improve a wide range of age-related cellular and functional impairments throughout the body. For example, frailty, which refers to a global decline in function that encompasses grip strength, activity, overall energy, and unintentional weight loss, is amenable to the effects of exercise ^17, 63, 64^. In rodent models, exercise reduces age-associated frailty through increasing skeletal muscle function ^63^ and has been shown to improve grip strength and nesting and burrowing behaviors even when initiated at middle age ^17^. In the normal aged brain, exercise has been shown to enhance regenerative capacity in aged mice by promoting hippocampal neurogenesis, effects that correlated with improved learning and memory ^65^. Furthermore, exercise also improved broad cellular hallmarks of brain aging in aged mice, including increased synaptic plasticity, improved neurovascular integrity, and decreased microglia activation ^17, 66, 67^. In the context of neurodegenerative disease, researchers have utilized mouse models of Alzheimer's disease to demonstrate that exercise is able to suppress inflammatory cytokines, increase the expression of antioxidant enzymes, improve synaptic function in the hippocampus, and enhance hippocampal-dependent learning and memory ^68^. Researchers have further explored the relationship between physical exercise, healthspan, and cognitive function in human populations. Epidemiological studies demonstrate that physical activity is associated with better overall survival and function in older adults compared to their sedentary counterparts ^69, 70^. It has been proposed that such benefits in elderly humans may be the result of counteracting muscle weakness and frailty as well as reducing circulating inflammatory markers ^71, 72^, consistent with animal studies. Exercise is also reported to reduce risk factors for cardiovascular disease, such as hypertension and metabolic syndrome ^71^, as well as reduce the risk of mild cognitive impairment later in life ^73– 76^. From a neurodegenerative disease perspective, a 6-month program of physical exercise in adults over the age of 50 years who are at risk for Alzheimer's disease also showed significantly improved cognition over an 18-month follow-up period ^77^. While the potential for human application exists, limitations should be noted, with evidence in humans indicating that the perception of physical frailty or poor health alone can decrease adherence in the elderly ^78^. Notwithstanding, these studies point to the capacity of exercise to extend healthspan and rejuvenate cellular and functional hallmarks of brain aging, with direct relevance to neurodegenerative diseases, such as Alzheimer’s disease.

### Parabiosis

Recently, the classical model of heterochronic parabiosis has re-emerged as an experimental platform to explore the intricate interplay between the systemic environment and organismal aging. To date, heterochronic parabiosis experiments have been used to explore the effects of aging throughout the body in tissues including muscle, pancreas, bone, heart, and brain ^35– 38, 40, 42, 79, 80^. Consistent with beneficial effects observed with exercise, the exposure of an aged mouse to a youthful systemic environment through heterochronic parabiosis likewise rejuvenates muscle function through decreased fibrogenic potential and improved regenerative capacity ^36– 38^. Moreover, heterochronic parabiosis also reversed cardiac hypertrophy and restored bone healing capacity in aged mice ^35, 42^. Excitingly, rejuvenating effects of heterochronic parabiosis are observed in the aged brain, indicating blood-borne mechanisms can counteract cellular and functional age-related neuronal decline ^39, 79– 81^.

## Systemic interventions: old blood and brain aging

Heterochronic parabiosis studies have also pointed to a role for old blood in driving brain aging ^79^. Regenerative capacity in young heterochronic parabionts is impaired, with neurogenesis decreasing in both the hippocampus and the subventricular zone neurogenic niches after exposure to old blood ^79, 80^. Interestingly, the detrimental effects of old blood observed in young heterochronic parabionts are specific to blood derived from old, but not middle-aged, animals ^80^. These data begin to tease apart the kinetics of circulating factors in old blood, defining ages at which to target pro-aging factors as a therapeutic approach. An independent approach to investigate the effects of blood exchange was recently developed using a microfluidic-based blood exchange device in rodents ^82^. With the use of this device, mice received a single heterochronic blood transfusion with whole blood derived from young or old mice, equivalent to 50% of total blood volume. Short-term exposure to old blood corroborated previous observations in the heterochronic parabiosis model that show an aged systemic environment negatively affects hippocampal adult neurogenesis. Further evidence of the pro-aging effects of old blood come from direct systemic interventions with old blood plasma injections. Short-term systemic administration of old plasma recapitulates the effects of heterochronic parabiosis on adult neurogenesis, implicating circulating factors in the effects of old blood ^82^. Moreover, long-term administration of old plasma over one month also resulted in impaired hippocampal-dependent learning and memory ^79^.

Thus far, only a few isolated circulating factors have been identified to mediate the effects of old blood. For example, the pleiotropic cytokine transforming growth factor beta (TGF-β) was identified to increase with age in both the circulation and the brain ^83^. Inhibition of TGF-β signaling systemically and locally by RNA interference or by pharmacological inhibition with TGF-β receptor kinase Alk5 inhibitor enhanced neurogenesis in the aged hippocampus ^83^. Using a proteomics-based approach in combination with heterochronic parabiosis, additional immune-related systemic pro-aging factors have been identified ^79^. In particular, the C-C motif chemokine 11 (CCL11) and beta-2 microglobulin (B2M) have been shown to negatively regulate neurogenesis and cognitive function in the hippocampus ^79, 81^. Moreover, using genetic knockout studies, researchers have shown that the loss of B2M prevents, in part, the aging-associated decline in neurogenesis and cognitive function at old age ^81^. Altogether, these results open up the possibility that functional rejuvenation in the aged brain may be possible by targeting specific circulating factors in old blood, potentially through interventions such as small-molecule-mediated pharmacological inhibition or the administration of neutralizing antibodies.

## Systemic interventions: young blood and brain rejuvenation

Evaluation of brain aging hallmarks in old heterochronic parabionts has further bolstered our appreciation for the inherent plasticity of the aged brain. Notably, regenerative capacity in the aged hippocampus and subventricular zone of heterochronic parabionts was enhanced after exposure to a young systemic environment ^39, 80^. This rejuvenating potential was maintained at a cell autonomous level with neural stem cells from old heterochronic parabionts showing enhanced self-renewal potential *in vitro* ^80^. Vascularization of the neurogenic niche is known to influence neural stem cell function ^84, 85^, and correspondingly heterochronic parabiosis also restored blood vessel volume in the aged subventricular zone neurogenic niche to youthful levels ^80^. Beyond regenerative function, exposure to a young systemic environment also enhanced synaptic plasticity, eliciting an increase in the expression of immediate early genes and the density of dendritic spines as well as enhancements in LTP ^39^. At a cognitive level, long-term administration of young plasma over one month was sufficient to reverse cognitive impairments in hippocampal-dependent learning and memory in old mice through increased activation of the transcription factor cAMP response element binding protein (CREB) ^39^. Consistently, old heterochronic parabionts that were separated from their young partner also demonstrated improvements in their olfactory discrimination ability compared to separated old isochronic controls. In a pre-clinical experiment, human umbilical cord-blood-derived plasma was demonstrated to similarly enhance immediate early gene expression, increase LTP, and improve cognitive function in aged mice ^86^. This demonstration of the rejuvenating potential of human umbilical cord blood in mice strengthens the possibility that human blood can also be used to elicit brain rejuvenation in humans.

Thus far, growth differentiation factor 11 (GDF11), colony-stimulating factor 2 (CSF2), and tissue inhibitor of metalloproteinases 2 (TIMP2) have been identified as rejuvenating factors in young adult and/or juvenile blood ^35, 86^. While its role in cardiac and skeletal muscle rejuvenation is currently under discussion ^35, 38, 87^, GDF11 was demonstrated to enhance neurogenesis and cerebral blood flow in aged mice when systemically administered ^80^. Hinting at the multifactorial nature of young plasma, TIMP2 or CSF2 administration was also shown to enhance immediate early gene expression, LTP, and cognitive performance in aged mice ^86^. Despite their shared rejuvenating effects, GDF11 was identified in young adult mouse plasma, while TIMP2 and CSF2 were identified in human umbilical cord blood, a comparatively early developmental stage. These distinctions again point to the importance of kinetics in determining circulating factor changes ^35, 86^. As more insight into the rejuvenating potential of young blood is obtained, it becomes evident that research is necessary to identify a breadth of factors by which to reverse global hallmarks of brain aging. While the burgeoning field of rejuvenation research is fast growing, and mounting evidence supports the translation of these interventions to humans, fundamental questions pertinent to therapeutic applications remain unexplored—in particular, how long lasting are the rejuvenating effects of young blood in the aged brain?

## Systemic interventions: young blood and neurodegenerative disease

Current neurodegenerative disease animal models recapitulate many pathologies observed in humans, including amyloid plaque deposition, tau phosphorylation, increased neuroinflammation, altered synaptic plasticity, and cognitive impairments ^88^. Despite their success in dissecting cellular and molecular changes involved in disease progression, current models are limited by the increasing array of transgenes they rely on, complicating interpretations and translation to human disease ^88^. Additionally, such brain-centric models have also precluded the possibility of investigating the systemic contribution to neurodegenerative disease. This is especially limiting when one considers the number of systemic diseases, such as diabetes and atherosclerosis, for which neurodegeneration is a common co-morbidity ^89^. Nevertheless, combining neurodegenerative disease animal models with systemic interventions, such as heterochronic parabiosis, has yielded promising results indicating the potential of young blood to counteract a number of neurodegenerative pathologies ( Figure 1).

To date, young blood studies have focused on animal models of Alzheimer’s disease. Changes in Alzheimer’s disease-related pathology were first investigated in parabiosis studies in which young transgenic mice carrying the human amyloid precursor protein ( *APP*) gene containing Swedish mutations and the human presenilin ( *PS1*) gene encoding the deleted exon 9 mutation (APPswe/PS1dE9) were joined with age-matched young wild-type animals ^90^. Exposure to a young wild-type systemic environment resulted in decreased plaque accumulation in APPswe/PS1dE9 transgenic mice after six months of parabiosis ^90^. Additionally, pro-inflammatory cytokines, levels of tau phosphorylation, and gliosis were reduced in transgenic mice following exposure to a young wild-type circulatory system ^90^. While this was not a direct effect of rejuvenation, as all parabionts were age-matched isochronic pairs, it does suggest a significant role for the systemic environment in the progression of neurodegenerative disease. Correspondingly, the use of peritoneal dialysis to filter amyloid-beta from the blood of APPswe/PS1dE9 transgenic mice also ameliorated Alzheimer’s-associated phenotypes, further demonstrating a role for the systemic environment in disease pathogenesis ^91^. More recently, the effect of young blood in the context of aging was investigated using heterochronic parabiosis in a complementary transgenic mouse model of Alzheimer’s disease carrying the human *APP* gene harboring human familial London and Swedish mutations ^92^. This study demonstrated increased expression of the synaptic marker synaptophysin and the pro-survival calcium binding protein calbindin in aged APP heterochronic parabionts exposed to a young systemic environment ^92^. No changes in amyloid plaque deposition or microglia activation were observed ^92^. Failure of heterochronic parabiosis to reverse plaque deposition and neuroinflammation after significant disease progression at old age suggests that it may be necessary to initiate systemic interventions prior to significant disease progression. Additionally, the duration of parabiosis used in the two studies above differed greatly from a more long-term six-month duration ^90^ to a shorter five-week duration ^92^, indicating that different disease pathologies may also prove amenable to improvements at vastly different timeframes. Lastly, at a cognitive level, injections of young wild-type plasma into aged APP transgenic mice also elicited improvements in hippocampal-dependent learning and memory ^92^. Of note, the benefits of administering specific pro-youthful factors in neurodegenerative disease models have yet to be tested. However, treatment with neurotrophic compounds has previously been demonstrated to attenuate neurodegenerative disease pathology in a triple-transgenic Alzheimer’s disease mouse model harboring human APPswe, PS1, and tau mutations ^93, 94^. These emerging studies raise significant translational potential for blood-based therapeutic approaches to counter neurodegenerative disease progression at a functional and cognitive level.

## Blood-based clinical trials for dementia-related neurodegenerative disease

Systemic interventions, including CR and exercise, have been previously evaluated in clinical trials, demonstrating beneficial effects on human healthspan and cognition (reviewed above). Notwithstanding, physical and technical barriers to adherence remain in the therapeutic application of exercise and CR. Coupled with promising results from young blood studies in animal models, particularly the recent demonstration of the rejuvenating effects of human umbilical cord plasma, researchers are now working to translate alternative blood-based systemic interventions to the clinic. Currently, a handful of ongoing clinical trials are seeking to identify biomarkers of healthspan and cognitive decline as well as exploring the potential of blood-based therapeutic interventions for the treatment of dementia-related neurodegenerative disease.

The Genetic and Epigenetic Signatures of Translational Aging Laboratory Testing (GESTALT) trial has taken a holistic approach to identifying biomarkers of healthspan ^95^. The researchers aim to correlate biomarkers from blood, muscle, and skin with performance in a range of physical and cognitive exams for a 10-year period in healthy adults over the age of 20. They will assess peripheral blood mononuclear cells compared to muscle and skin biomarker data to assess systemic versus tissue-derived age-related changes and investigate relationships between changes in biomarker levels and hallmarks of aging, such as cognitive decline and increased inflammation. A second trial run by the Stanford Memory and Aging Study aims to identify proteins in blood and cerebrospinal fluid (CSF) of healthy older adults (60–90 years old) that correlate with changes from magnetic resonance imaging (MRI), neuropsychological and neurological testing, and memory performance ^96^. Stanford University is also conducting a longitudinal study, the Healthy Brain Aging Study, that seeks to identify blood and CSF biomarkers associated with MRI and cognitive testing in patients with dementia-related neurodegenerative diseases ^97^. Uniquely, this study aims to follow enrollees over time, ending in eventual brain donation to obtain a more complete assessment of disease progression. Collectively, these biomarker studies will be crucial in identifying potential indicators of human aging and early development of neurodegenerative disease-related cognitive decline.

To explore the translational potential of young plasma as a treatment for neurodegenerative disease-related cognitive decline, two major studies were independently initiated. The first study, PLasma for Alzheimer SymptoM Amelioration (PLASMA), was initiated in 2014 by Stanford University in collaboration with Alkahest, a company based in San Carlos, California ^98^. This study enrolled patients with mild-to-moderate Alzheimer's disease for infusions of one unit of plasma derived from males aged 30 years or younger once weekly for four weeks. While the primary outcome is to determine the feasibility and safety of young plasma administration, the researchers will assess memory performance, psychological status, and MRI analysis. Concurrently, they will analyze plasma levels of factors previously associated with neurodegenerative disease. The second study, Young Donor Plasma Transfusion of Age-Related Biomarkers, is being conducted by the company Ambrosia, a startup based in Monterey, California ^99^. In contrast to the PLASMA study, enrollees in the Ambrosia trial can be healthy or disease-affected individuals over 35 years of age. Enrollees will receive a single infusion of plasma from young donors aged 16–25. Researchers will then assess a specific panel of plasma factors previously associated with aging and neurodegenerative hallmarks, such as inflammation, neurogenesis, and amyloid plaque deposition. Of note, a number of concerns have been raised with the design of the Ambrosia-led study, including the omission of an independent placebo control group ^100^. Lastly, the potential of using young plasma interventions is also being explored in a broader context of neurodegenerative disease, including Parkinson's disease ^101^, progressive supranuclear palsy ^102^, and acute stroke ^103^. While any of these studies has yet to yield results, the outcomes from the young plasma administration trials, coupled with the perspective gained from the extensive biomarker studies, will provide a more complete picture of potential pro-aging and rejuvenating circulating factors that can be targeted to increase healthspan and ameliorate neurodegenerative disease in the elderly.

## Conclusion

Systemic interventions, such as CR, exercise, and young blood administration, have reliably ameliorated many facets of functional and cognitive decline in rodent models of aging and neurodegenerative disease by targeting molecular and cellular pathways conserved across species ( Figure 1). Collectively, the rejuvenating power of these systemic interventions has peaked interest in the public at large for their potential therapeutic translation to humans. Coupled with the most recent reports that human cord-blood-derived plasma proteins can also counteract age-related cognitive impairments in aged mice, the possibility for brain rejuvenation strategies in humans is increasingly strengthened. To that end, multiple clinical trials are now underway to test the safety and efficacy of systemic brain rejuvenation strategies in the elderly under normal aging and neurodegenerative disease conditions. While current clinical trials have limitations, they are based on a strong foundation of animal research, with much reason for excitement in the potential to develop effective systemic strategies for the treatment of dementia-related neurodegenerative disease. Looking toward the future, barriers posed by the idiopathic nature of many neurodegenerative diseases, unknown mechanisms driving the rejuvenating effects of blood, and added inter-individual variability introduced by human subjects must now be overcome in the pursuit to fully tap into the therapeutic potential of systemic brain rejuvenation strategies. Additionally, remaining questions as to how pro-aging and rejuvenating factors in blood can be manipulated in concert to maximize the potential therapeutic benefit to patients affected by neurodegenerative disease must also be addressed, providing a rich scientific field of inquiry to explore. Broadly, future research investigating the potential for brain rejuvenation may not only impact human health but also yield fundamental understanding of the biological mechanisms governing the aging process itself.

## Abbreviations

APP, amyloid precursor protein; B2M, beta-2 microglobulin; CR, caloric restriction; CSF, cerebrospinal fluid; CSF2, colony-stimulating factor 2; GDF11, growth differentiation factor 11; LTP, long-term potentiation; MRI, magnetic resonance imaging; PS1, human presenilin 1; TGF-β, transforming growth factor beta; TIMP2, tissue inhibitor of metalloproteinases 2.

## References

[1] López-Otín C Blasco MA Partridge L: The hallmarks of aging. *Cell.* 2013;153(6):1194–217. 10.1016/j.cell.2013.05.039 23746838 PMC3836174

[2] Mather M Jacobsen LA Pollard KM: Aging in the United States.2015;70 Reference Source

[3] Alzheimer's Association: 2016 Alzheimer's disease facts and figures. *Alzheimers Dement.* 2016;12(4):459–509. 10.1016/j.jalz.2016.03.001 27570871

[4] Wyss-Coray T: Ageing, neurodegeneration and brain rejuvenation. *Nature.* 2016;539(7628):180–6. 10.1038/nature20411 27830812 PMC5172605

[5] Morrison JH Baxter MG: The ageing cortical synapse: hallmarks and implications for cognitive decline. *Nat Rev Neurosci.* 2012;13(4):240–50. 10.1038/nrn3200 22395804 PMC3592200

[6] Farkas E Luiten PG: Cerebral microvascular pathology in aging and Alzheimer's disease. *Prog Neurobiol.* 2001;64(6):575–611. 10.1016/S0301-0082(00)00068-X 11311463

[7] Conde JR Streit WJ: Microglia in the aging brain. *J Neuropathol Exp Neurol.* 2006;65(3):199–203. 10.1097/01.jnen.0000202887.22082.63 16651881

[8] Mattson MP Magnus T: Ageing and neuronal vulnerability. *Nat Rev Neurosci.* 2006;7(4):278–94. 10.1038/nrn1886 16552414 PMC3710114

[9] Kuhn HG Dickinson-Anson H Gage FH: Neurogenesis in the dentate gyrus of the adult rat: age-related decrease of neuronal progenitor proliferation. *J Neurosci.* 1996;16(6):2027–33. 8604047 10.1523/JNEUROSCI.16-06-02027.1996 PMC6578509

[10] Bondolfi L Ermini F Long JM: Impact of age and caloric restriction on neurogenesis in the dentate gyrus of C57BL/6 mice. *Neurobiol Aging.* 2004;25(3):333–40. 10.1016/S0197-4580(03)00083-6 15123339

[11] Kuipers SD Schroeder JE Trentani A: Changes in hippocampal neurogenesis throughout early development. *Neurobiol Aging.* 2015;36(1):365–79. 10.1016/j.neurobiolaging.2014.07.033 25172123

[12] Bond AM Ming GL Song H: Adult Mammalian Neural Stem Cells and Neurogenesis: Five Decades Later. *Cell Stem Cell.* 2015;17(4):385–95. 10.1016/j.stem.2015.09.003 26431181 PMC4683085

[13] Alvarez-Buylla A Garcia-Verdugo JM: Neurogenesis in adult subventricular zone. *J Neurosci.* 2002;22(3):629–34. 11826091 10.1523/JNEUROSCI.22-03-00629.2002 PMC6758521

[14] Drapeau E Mayo W Aurousseau C: Spatial memory performances of aged rats in the water maze predict levels of hippocampal neurogenesis. *Proc Natl Acad Sci U S A.* 2003;100(24):14385–90. 10.1073/pnas.2334169100 14614143 PMC283601

[15] Merrill DA Karim R Darraq M: Hippocampal cell genesis does not correlate with spatial learning ability in aged rats. *J Comp Neurol.* 2003;459(2):201–7. 10.1002/cne.10616 12640670

[16] Riddle DR Sonntag WE Lichtenwalner RJ: Microvascular plasticity in aging. *Ageing Res Rev.* 2003;2(2):149–68. 10.1016/S1568-1637(02)00064-8 12605958

[17] Soto I Graham LC Richter HJ: APOE Stabilization by Exercise Prevents Aging Neurovascular Dysfunction and Complement Induction. *PLoS Biol.* 2015;13(10):e1002279. 10.1371/journal.pbio.1002279 26512759 PMC4626092

[18] Hong S Beja-Glasser VF Nfonoyim BM: Complement and microglia mediate early synapse loss in Alzheimer mouse models. *Science.* 2016;352(6286):712–6. 10.1126/science.aad8373 27033548 PMC5094372

[19] Peters A Sethares C Luebke JI: Synapses are lost during aging in the primate prefrontal cortex. *Neuroscience.* 2008;152(4):970–81. 10.1016/j.neuroscience.2007.07.014 18329176 PMC2441531

[20] Selkoe DJ: Alzheimer's disease is a synaptic failure. *Science.* 2002;298(5594):789–91. 10.1126/science.1074069 12399581

[21] Geiszler PC Barron MR Pardon MC: Impaired burrowing is the most prominent behavioral deficit of aging htau mice. *Neuroscience.* 2016;329:98–111. 10.1016/j.neuroscience.2016.05.004 27167086 PMC4915442

[22] Murphy CT McCarroll SA Bargmann CI: Genes that act downstream of DAF-16 to influence the lifespan of *Caenorhabditis elegans*. *Nature.* 2003;424(6946):277–83. 10.1038/nature01789 12845331

[23] Lin YJ Seroude L Benzer S: Extended life-span and stress resistance in the *Drosophila* mutant *methuselah*. *Science.* 1998;282(5390):943–6. 10.1126/science.282.5390.943 9794765

[24] Satoh A Brace CS Rensing N: Sirt1 extends life span and delays aging in mice through the regulation of Nk2 homeobox 1 in the DMH and LH. *Cell Metab.* 2013;18(3):416–30. 10.1016/j.cmet.2013.07.013 24011076 PMC3794712

[25] Zhang G Li J Purkayastha S: Hypothalamic programming of systemic ageing involving IKK-β, NF-κB and GnRH. *Nature.* 2013;497(7448):211–6. 10.1038/nature12143 23636330 PMC3756938

[26] Ocampo A Reddy P Martinez-Redondo P: *In Vivo* Amelioration of Age-Associated Hallmarks by Partial Reprogramming. *Cell.* 2016;167(7):1719–1733.e12. 10.1016/j.cell.2016.11.052 27984723 PMC5679279

[27] Rahman MM Stuchlick O El-Karim EG: Intracellular protein glycosylation modulates insulin mediated lifespan in C.elegans. *Aging (Albany NY).* 2010;2(10):678–90. 10.18632/aging.100208 20952811 PMC2993798

[28] Harrison DE Strong R Sharp ZD: Rapamycin fed late in life extends lifespan in genetically heterogeneous mice. *Nature.* 2009;460(7253):392–5. 10.1038/nature08221 19587680 PMC2786175

[29] Ryu D Mouchiroud L Andreux PA: Urolithin A induces mitophagy and prolongs lifespan in *C. elegans* and increases muscle function in rodents. *Nat Med.* 2016;22(8):879–88. 10.1038/nm.4132 27400265

[30] Zhang H Ryu D Wu Y: NAD ^+^ repletion improves mitochondrial and stem cell function and enhances life span in mice. *Science.* 2016;352(6292):1436–43. 10.1126/science.aaf2693 27127236

[31] Baker DJ Childs BG Durik M: Naturally occurring p16 ^Ink4a^-positive cells shorten healthy lifespan. *Nature.* 2016;530(7589):184–9. 10.1038/nature16932 26840489 PMC4845101

[32] Goodrick CL: The effects of exercise on longevity and behavior of hybrid mice which differ in coat color. *J Gerontol.* 1974;29(2):129–33. 10.1093/geronj/29.2.129 4811947

[33] Ludwig FC Elashoff RM: Mortality in syngeneic rat parabionts of different chronological age. *Trans N Y Acad Sci.* 1972;34(7):582–7. 10.1111/j.2164-0947.1972.tb02712.x 4507935

[34] Weindruch R Walford RL: Dietary restriction in mice beginning at 1 year of age: effect on life-span and spontaneous cancer incidence. *Science.* 1982;215(4538):1415–8. 10.1126/science.7063854 7063854

[35] Loffredo FS Steinhauser ML Jay SM: Growth differentiation factor 11 is a circulating factor that reverses age-related cardiac hypertrophy. *Cell.* 2013;153(4):828–39. 10.1016/j.cell.2013.04.015 23663781 PMC3677132

[36] Conboy IM Conboy MJ Wagers AJ: Rejuvenation of aged progenitor cells by exposure to a young systemic environment. *Nature.* 2005;433(7027):760–4. 10.1038/nature03260 15716955

[37] Brack AS Conboy MJ Roy S: Increased Wnt signaling during aging alters muscle stem cell fate and increases fibrosis. *Science.* 2007;317(5839):807–10. 10.1126/science.1144090 17690295

[38] Sinha M Jang YC Oh J: Restoring systemic GDF11 levels reverses age-related dysfunction in mouse skeletal muscle. *Science.* 2014;344(6184):649–52. 10.1126/science.1251152 24797481 PMC4104429

[39] Villeda SA Plambeck KE Middeldorp J: Young blood reverses age-related impairments in cognitive function and synaptic plasticity in mice. *Nat Med.* 2014;20(6):659–63. 10.1038/nm.3569 24793238 PMC4224436

[40] Salpeter SJ Khalaileh A Weinberg-Corem N: Systemic regulation of the age-related decline of pancreatic β-cell replication. *Diabetes.* 2013;62(8):2843–8. 10.2337/db13-0160 23630298 PMC3717843

[41] Ruckh JM Zhao JW Shadrach JL: Rejuvenation of regeneration in the aging central nervous system. *Cell Stem Cell.* 2012;10(1):96–103. 10.1016/j.stem.2011.11.019 22226359 PMC3714794

[42] Baht GS Silkstone D Vi L: Exposure to a youthful circulation rejuvenates bone repair through modulation of β-catenin. *Nat Commun.* 2015;6: 7131. 10.1038/ncomms8131 25988592 PMC4479006

[43] Colman RJ Beasley TM Kemnitz JW: Caloric restriction reduces age-related and all-cause mortality in rhesus monkeys. *Nat Commun.* 2014;5: 3557. 10.1038/ncomms4557 24691430 PMC3988801

[44] Weindruch R Walford RL: The Retardation of aging and disease by dietary restriction. *J Nutr.* 1988 Reference Source 10.1093/jn/116.4.641 3958810

[45] Colman RJ Anderson RM Johnson SC: Caloric restriction delays disease onset and mortality in rhesus monkeys. *Science.* 2009;325(5937):201–4. 10.1126/science.1173635 19590001 PMC2812811

[46] Guo J Bakshi V Lin AL: Early Shifts of Brain Metabolism by Caloric Restriction Preserve White Matter Integrity and Long-Term Memory in Aging Mice. *Front Aging Neurosci.* 2015;7:213. 10.3389/fnagi.2015.00213 26617514 PMC4643125

[47] Mattson MP: Neuroprotective signaling and the aging brain: take away my food and let me run. *Brain Res.* 2000;886(1–2):47–53. 10.1016/S0006-8993(00)02790-6 11119686

[48] Ferreira-Marques M Aveleira CA Carmo-Silva S: Caloric restriction stimulates autophagy in rat cortical neurons through neuropeptide Y and ghrelin receptors activation. *Aging (Albany NY).* 2016;8(7):1470–84. 10.18632/aging.100996 27441412 PMC4993343

[49] Van Cauwenberghe C Vandendriessche C Libert C: Caloric restriction: beneficial effects on brain aging and Alzheimer's disease. *Mamm Genome.* 2016;27(7–8):300–19. 10.1007/s00335-016-9647-6 27240590

[50] Lin AL Zhang W Gao X: Caloric restriction increases ketone bodies metabolism and preserves blood flow in aging brain. *Neurobiol Aging.* 2015;36(7):2296–303. 10.1016/j.neurobiolaging.2015.03.012 25896951 PMC4457572

[51] Parikh I Guo J Chuang KH: Caloric restriction preserves memory and reduces anxiety of aging mice with early enhancement of neurovascular functions. *Aging (Albany NY).* 2016;8(11):2814–26. 10.18632/aging.101094 27829242 PMC5191872

[52] Villain N Picq JL Aujard F: Body mass loss correlates with cognitive performance in primates under acute caloric restriction conditions. *Behav Brain Res.* 2016;305:157–63. 10.1016/j.bbr.2016.02.037 26952885

[53] Dal-Pan A Pifferi F Marchal J: Cognitive performances are selectively enhanced during chronic caloric restriction or resveratrol supplementation in a primate. *PLoS One.* 2011;6(1):e16581. 10.1371/journal.pone.0016581 21304942 PMC3031601

[54] Ma L Zhao Z Wang R: Caloric restriction can improve learning ability in C57/BL mice via regulation of the insulin-PI3K/Akt signaling pathway. *Neurol Sci.* 2014;35(9):1381–6. 10.1007/s10072-014-1717-5 24651932

[55] Solon-Biet SM Cogger VC Pulpitel T: Defining the Nutritional and Metabolic Context of FGF21 Using the Geometric Framework. *Cell Metab.* 2016;24(4):555–65. 10.1016/j.cmet.2016.09.001 27693377

[56] Martin SA DeMuth TM Miller KN: Regional metabolic heterogeneity of the hippocampus is nonuniformly impacted by age and caloric restriction. *Aging Cell.* 2016;15(1):100–10. 10.1111/acel.12418 26521867 PMC4717265

[57] Cardoso A Marrana F Andrade JP: Caloric restriction in young rats disturbs hippocampal neurogenesis and spatial learning. *Neurobiol Learn Mem.* 2016;133:214–24. 10.1016/j.nlm.2016.07.013 27432519

[58] Rühlmann C Wölk T Blümel T: Long-term caloric restriction in *ApoE*-deficient mice results in neuroprotection via Fgf21-induced AMPK/mTOR pathway. *Aging (Albany NY).* 2016;8(11):2777–89. 10.18632/aging.101086 27902456 PMC5191869

[59] Halagappa VK Guo Z Pearson M: Intermittent fasting and caloric restriction ameliorate age-related behavioral deficits in the triple-transgenic mouse model of Alzheimer's disease. *Neurobiol Dis.* 2007;26(1):212–20. 10.1016/j.nbd.2006.12.019 17306982

[60] Ravussin E Redman LM Rochon J: A 2-Year Randomized Controlled Trial of Human Caloric Restriction: Feasibility and Effects on Predictors of Health Span and Longevity. *J Gerontol A Biol Sci Med Sci.* 2015;70(9):1097–104. 10.1093/gerona/glv057 26187233 PMC4841173

[61] Navarro A Gomez C López-Cepero JM: Beneficial effects of moderate exercise on mice aging: survival, behavior, oxidative stress, and mitochondrial electron transfer. *Am J Physiol Regul Integr Comp Physiol.* 2004;286(3):R505–11. 10.1152/ajpregu.00208.2003 14615275

[62] Samorajski T Delaney C Durham L: Effect of exercise on longevity, body weight, locomotor performance, and passive-avoidance memory of C57BL/6J mice. *Neurobiol Aging.* 1985;6(1):17–24. 10.1016/0197-4580(85)90066-1 4000382

[63] Garcia-Valles R Gomez-Cabrera MC Rodriguez-Mañas L: Life-long spontaneous exercise does not prolong lifespan but improves health span in mice. *Longev Healthspan.* 2013;2(1):14. 10.1186/2046-2395-2-14 24472376 PMC3922914

[64] Xue QL: The frailty syndrome: definition and natural history. *Clin Geriatr Med.* 2011;27(1):1–15. 10.1016/j.cger.2010.08.009 21093718 PMC3028599

[65] van Praag H Shubert T Zhao C: Exercise enhances learning and hippocampal neurogenesis in aged mice. *J Neurosci.* 2005;25(38):8680–5. 10.1523/JNEUROSCI.1731-05.2005 16177036 PMC1360197

[66] Speisman RB Kumar A Rani A: Daily exercise improves memory, stimulates hippocampal neurogenesis and modulates immune and neuroimmune cytokines in aging rats. *Brain Behav Immun.* 2013;28:25–43. 10.1016/j.bbi.2012.09.013 23078985 PMC3545095

[67] O'Callaghan RM Griffin EW Kelly AM: Long-term treadmill exposure protects against age-related neurodegenerative change in the rat hippocampus. *Hippocampus.* 2009;19(10):1019–29. 10.1002/hipo.20591 19309034

[68] Intlekofer KA Cotman CW: Exercise counteracts declining hippocampal function in aging and Alzheimer's disease. *Neurobiol Dis.* 2013;57:47–55. 10.1016/j.nbd.2012.06.011 22750524

[69] Stessman J Hammerman-Rozenberg R Cohen A: Physical activity, function, and longevity among the very old. *Arch Intern Med.* 2009;169(16):1476–83. 10.1001/archinternmed.2009.248 19752405

[70] Chakravarty EF Hubert HB Lingala VB: Reduced disability and mortality among aging runners: a 21-year longitudinal study. *Arch Intern Med.* 2008;168(15):1638–46. 10.1001/archinte.168.15.1638 18695077 PMC3175643

[71] Colbert LH Visser M Simonsick EM: Physical activity, exercise, and inflammatory markers in older adults: findings from the Health, Aging and Body Composition Study. *J Am Geriatr Soc.* 2004;52(7):1098–104. 10.1111/j.1532-5415.2004.52307.x 15209647

[72] Fiatarone MA O'Neill EF Ryan ND: Exercise training and nutritional supplementation for physical frailty in very elderly people. *N Engl J Med.* 1994;330(25):1769–75. 10.1056/NEJM199406233302501 8190152

[73] Kirk-Sanchez NJ McGough EL: Physical exercise and cognitive performance in the elderly: current perspectives. *Clin Interv Aging.* 2014;9:51–62. 10.2147/CIA.S39506 24379659 PMC3872007

[74] Geda YE Roberts RO Knopman DS: Physical exercise, aging, and mild cognitive impairment: a population-based study. *Arch Neurol.* 2010;67(1):80–6. 10.1001/archneurol.2009.297 20065133 PMC2919839

[75] Knecht S Wersching H Lohmann H: High-normal blood pressure is associated with poor cognitive performance. *Hypertension.* 2008;51(3):663–8. 10.1161/HYPERTENSIONAHA.107.105577 18250360

[76] Fujishima M Ibayashi S Fujii K: Cerebral blood flow and brain function in hypertension. *Hypertens Res.* 1995;18(2):111–7. 10.1291/hypres.18.111 7584916

[77] Lautenschlager NT Cox KL Flicker L: Effect of physical activity on cognitive function in older adults at risk for Alzheimer disease: a randomized trial. *JAMA.* 2008;300(9):1027–37. 10.1001/jama.300.9.1027 18768414

[78] Rhodes RE Martin AD Taunton JE: Factors associated with exercise adherence among older adults. An individual perspective. *Sports Med.* 1999;28(6):397–411. 10.2165/00007256-199928060-00003 10623983

[79] Villeda SA Luo J Mosher KI: The ageing systemic milieu negatively regulates neurogenesis and cognitive function. *Nature.* 2011;477(7362):90–4. 10.1038/nature10357 21886162 PMC3170097

[80] Katsimpardi L Litterman NK Schein PA: Vascular and neurogenic rejuvenation of the aging mouse brain by young systemic factors. *Science.* 2014;344(6184):630–4. 10.1126/science.1251141 24797482 PMC4123747

[81] Smith LK He Y Park JS: β2-microglobulin is a systemic pro-aging factor that impairs cognitive function and neurogenesis. *Nat Med.* 2015;21(8):932–7. 10.1038/nm.3898 26147761 PMC4529371

[82] Rebo J Mehdipour M Gathwala R: A single heterochronic blood exchange reveals rapid inhibition of multiple tissues by old blood. *Nat Commun.* 2016;7: 13363. 10.1038/ncomms13363 27874859 PMC5121415

[83] Yousef H Conboy MJ Morgenthaler A: Systemic attenuation of the TGF-β pathway by a single drug simultaneously rejuvenates hippocampal neurogenesis and myogenesis in the same old mammal. *Oncotarget.* 2015;6(14):11959–78. 10.18632/oncotarget.3851 26003168 PMC4494916

[84] Tavazoie M van der Veken L Silva-Vargas V: A specialized vascular niche for adult neural stem cells. *Cell Stem Cell.* 2008;3(3):279–88. 10.1016/j.stem.2008.07.025 18786415 PMC6864413

[85] Shen Q Goderie SK Jin L: Endothelial cells stimulate self-renewal and expand neurogenesis of neural stem cells. *Science.* 2004;304(5675):1338–40. 10.1126/science.1095505 15060285

[86] Castellano JM Mosher KI Abbey RJ: Human umbilical cord plasma proteins revitalize hippocampal function in aged mice. *Nature.* 2017;544(7651):488–92. 10.1038/nature22067 28424512 PMC5586222

[87] Egerman MA Cadena SM Gilbert JA: GDF11 Increases with Age and Inhibits Skeletal Muscle Regeneration. *Cell Metab.* 2015;22(1):164–74. 10.1016/j.cmet.2015.05.010 26001423 PMC4497834

[88] Jucker M: The benefits and limitations of animal models for translational research in neurodegenerative diseases. *Nat Med.* 2010;16(11):1210–4. 10.1038/nm.2224 21052075

[89] Cunningham C Hennessy E: Co-morbidity and systemic inflammation as drivers of cognitive decline: new experimental models adopting a broader paradigm in dementia research. *Alzheimers Res Ther.* 2015;7(1):33. 10.1186/s13195-015-0117-2 25802557 PMC4369837

[90] Xiang Y Bu XL Liu YH: Physiological amyloid-beta clearance in the periphery and its therapeutic potential for Alzheimer's disease. *Acta Neuropathol.* 2015;130(4):487–99. 10.1007/s00401-015-1477-1 26363791 PMC4575389

[91] Jin WS Shen LL Bu XL: Peritoneal dialysis reduces amyloid-beta plasma levels in humans and attenuates Alzheimer-associated phenotypes in an APP/PS1 mouse model. *Acta Neuropathol.* 2017;134(2):207–220. 10.1007/s00401-017-1721-y 28477083

[92] Middeldorp J Lehallier B Villeda SA: Preclinical Assessment of Young Blood Plasma for Alzheimer Disease. *JAMA Neurol.* 2016;73(11):1325–33. 10.1001/jamaneurol.2016.3185 27598869 PMC5172595

[93] Kazim SF Blanchard J Dai CL: Disease modifying effect of chronic oral treatment with a neurotrophic peptidergic compound in a triple transgenic mouse model of Alzheimer's disease. *Neurobiol Dis.* 2014;71:110–30. 10.1016/j.nbd.2014.07.001 25046994

[94] Baazaoui N Iqbal K: Prevention of Amyloid-β and Tau Pathologies, Associated Neurodegeneration, and Cognitive Deficit by Early Treatment with a Neurotrophic Compound. *J Alzheimers Dis.* 2017;58(1):215–30. 10.3233/JAD-170075 28387677

[95] Ferrucci L National Institute of Aging: Genetic and Epigenetic Signatures of Translational Aging Laboratory Testing (GESTALT). In: ClinicalTrials.gov. [cited 2017 Apr 10]. Reference Source

[96] Wagner A: Stanford University, Stanford Memory and Aging Study.In: Nia.nih.gov. [cited 2017 Apr 10]. Reference Source

[97] Henderson V Wyss-Cora T: Stanford University, Healthy Brain Aging Study.In : https://med.stanford.edu/[cited 2017 Apr 10]. Reference Source

[98] Stanford University, Alkahest, Inc.,: The PLasma for Alzheimer SymptoM Amelioration (PLASMA) Study. In: ClinicalTrials.gov. [cited 2017 Apr 10]. Reference Source

[99] Karmazin J Ambrosia LLC: Young Donor Plasma Transfusion and Age-Related Biomarkers.In: ClinicalTrials.gov. [cited 2017 Apr 10].2017 Reference Source

[100] Kaiser J: Young blood antiaging trial raises questions. *Science.* 2016 10.1126/science.aag0716 27493159

[101] Bronte-Stewart H: Stanford University, The Stanford Parkinson's Disease Plasma Study (SPDP). In: ClinicalTrials.gov. [cited 2017 Apr 10].2017 Reference Source

[102] Tsai R University of California, San Francisco: Young Plasma Transfusions for Progressive Supranuclear Palsy.In: ClinicalTrials.gov. [cited 2017 Apr 10].2017 Reference Source

[103] Xinqiao Hospital of Chongqing: Efficacy and Safety of Young Plasma on Acute Stroke.In ClinicalTrials.gov. [cited 2017 Apr 10]. Reference Source

